# CRISPR/Cas9-mediated gene knockout reveals a guardian role of NF-κB/RelA in maintaining the homeostasis of human vascular cells

**DOI:** 10.1007/s13238-018-0560-5

**Published:** 2018-07-02

**Authors:** Ping Wang, Zunpeng Liu, Xiaoqian Zhang, Jingyi Li, Liang Sun, Zhenyu Ju, Jian Li, Piu Chan, Guang-Hui Liu, Weiqi Zhang, Moshi Song, Jing Qu

**Affiliations:** 1 National Laboratory of Biomacromolecules, CAS Center for Excellence in Biomacromolecules, Institute of Biophysics, Chinese Academy of Sciences, Beijing, 100101 China; 2 State Key Laboratory of Stem Cell and Reproductive Biology, Institute of Zoology, Chinese Academy of Sciences, Beijing, 100101 China; 3 State Key Laboratory of Membrane Biology, Institute of Zoology, Chinese Academy of Sciences, Beijing, 100101 China; 4 University of Chinese Academy of Sciences, Beijing, 100049 China; 5 National Clinical Research Center for Geriatric Disorders, Xuanwu Hospital of Capital Medical University, Beijing, 100053 China; 6 Institute of Stem cell and Regeneration, Chinese Academy of Sciences, Beijing, 100101 China; 7 The MOH Key Laboratory of Geriatrics, Beijing Hospital, National Center of Gerontology, Beijing, 100730 China; 8 Key Laboratory of Regenerative Medicine of Ministry of Education, Institute of Aging and Regenerative Medicine, Jinan University, Guangzhou, 510632 China

**Keywords:** NF-κB, RelA, Stem cell, Inflammation, Apoptosis

## Abstract

Vascular cell functionality is critical to blood vessel homeostasis. Constitutive NF-κB activation in vascular cells results in chronic vascular inflammation, leading to various cardiovascular diseases. However, how NF-κB regulates human blood vessel homeostasis remains largely elusive. Here, using CRISPR/Cas9-mediated gene editing, we generated *RelA* knockout human embryonic stem cells (hESCs) and differentiated them into various vascular cell derivatives to study how NF-κB modulates human vascular cells under basal and inflammatory conditions. Multi-dimensional phenotypic assessments and transcriptomic analyses revealed that RelA deficiency affected vascular cells via modulating inflammation, survival, vasculogenesis, cell differentiation and extracellular matrix organization in a cell type-specific manner under basal condition, and that RelA protected vascular cells against apoptosis and modulated vascular inflammatory response upon tumor necrosis factor α (TNFα) stimulation. Lastly, further evaluation of gene expression patterns in *IκB*α knockout vascular cells demonstrated that IκBα acted largely independent of RelA signaling. Taken together, our data reveal a protective role of NF-κB/RelA in modulating human blood vessel homeostasis and map the human vascular transcriptomic landscapes for the discovery of novel therapeutic targets.

**Electronic supplementary material:**

The online version of this article (10.1007/s13238-018-0560-5) contains supplementary material, which is available to authorized users.

## Introduction

Blood vessels carry nutrients and oxygen via blood stream throughout the body. Blood vessel homeostasis is critical to health. Disorders of blood vessels impair vascular cell function, resulting in the pathogenesis of severe cardiovascular diseases like myocardial infarction, atherosclerosis, and stroke.

Blood vessels consist of three layers, namely the tunica intima, tunica media, and tunica adventitia. The major cell types of these three layers are vascular endothelial cells (VECs), vascular smooth muscle cells (VSMCs), and mesenchymal stem cells (MSCs), respectively. VECs compose a monolayer of cells called endothelium that modulates nutrients transport, vascular tone, host defense, homeostasis and angiogenesis (Galley and Webster, 2004). VSMCs constitute the majority of blood vessels and regulate local blood pressure via vasoconstriction and vasodilation. MSCs are part of the adventitia that modulates vascular inflammatory response, trophic supply, and vessel damage repair (Breitbach et al., 2018; Caplan and Correa, 2011; da Silva Meirelles et al., 2008). Vascular dysfunction manifests various cellular abnormalities like neointimal growth, thrombosis, inflammation, and vascular apoptosis and further leads to ischemia and infarction, thus providing prognostic parameters of cardiovascular diseases (Nedeljkovic et al., 2003; Zhang et al., 2018).

NF-κB is a transcription factor that modulates inflammatory response apart from regulating proliferation and survival (Perkins and Gilmore, 2006). In most cell types, the predominant form of NF-κB is a heterodimer composed of RelA/p65 and p50, which can be activated by various stimuli such as TNFα and IL1β (Tilstra et al., 2011). NF-κB is sequestered in the cytoplasm in an inactive complex bound to the inhibitory proteins, inhibitors of NF-κB (IκBs). Upon stimulations, the degradation of IκB releases NF-κB, which subsequently translocates to the nucleus to modulate genes for the maintenance of cellular homeostasis (Liu et al., 2008; Perkins, 2007). Previous studies have shown that NF-κB activation is present in atherosclerotic lesion, resulting in chemokine secretion, adhesion of circulating monocytes to endothelium and persistent inflammatory response in VECs (Baker et al., 2011; Brand et al., 1996; Hajra et al., 2000). In VSMCs, NF-κB activation promotes extracellular matrix expression, neointimal proliferation, aberrant inflammatory response, and cell apoptosis, leading to vascular restenosis and plaque rupture (Rudijanto, 2007). Conversely, inhibition of NF-κB in vascular cells often exhibits therapeutic effects on cardiovascular diseases, such as the amelioration of vascular atherosclerotic lesion in apolipoprotein E (ApoE)-deficient mice (Chiba et al., 2006; Gareus et al., 2008; Mallavia et al., 2013). However, little has been illustrated how NF-κB regulates the physiological functions of different human vascular cells.

Here, using CRISPR/Cas9-mediated genome editing, we generated RelA-deficient human embryonic stem cells (ESCs) to abolish NF-κB activity. We obtained RelA-deficient VECs, VSMCs and MSCs via directed differentiation and uncovered a protective role of NF-κB/RelA in different human vascular cells under basal and inflammatory conditions. RelA deficiency in VECs resulted in impaired vasculogenesis and monocyte-endothelium adhesion, as well as excessive proliferation. In MSCs, loss of RelA resulted in defective proliferation and dysregulated differentiation potential. Additionally, RelA deficiency promoted TNFα-induced apoptosis in different human vascular cells. Further transcriptomic analysis of RelA-deficient vascular cells revealed the cellular changes in vascular matrix organization, angiogenesis, inflammatory response, cell proliferation and survival. Lastly, analysis of gene expression patterns in various *IκB*α knockout vascular cells showed that IκBα acted largely independent of RelA signaling under basal condition and upon TNFα stimulation. Taken together, our data illustrate a novel protective role of NF-κB/RelA in human vascular cells and provide a platform for the study of NF-κB function in human adult stem cells and somatic cells.

## Results

### Generation of RelA-deficient human ESCs

**Figure 1:**
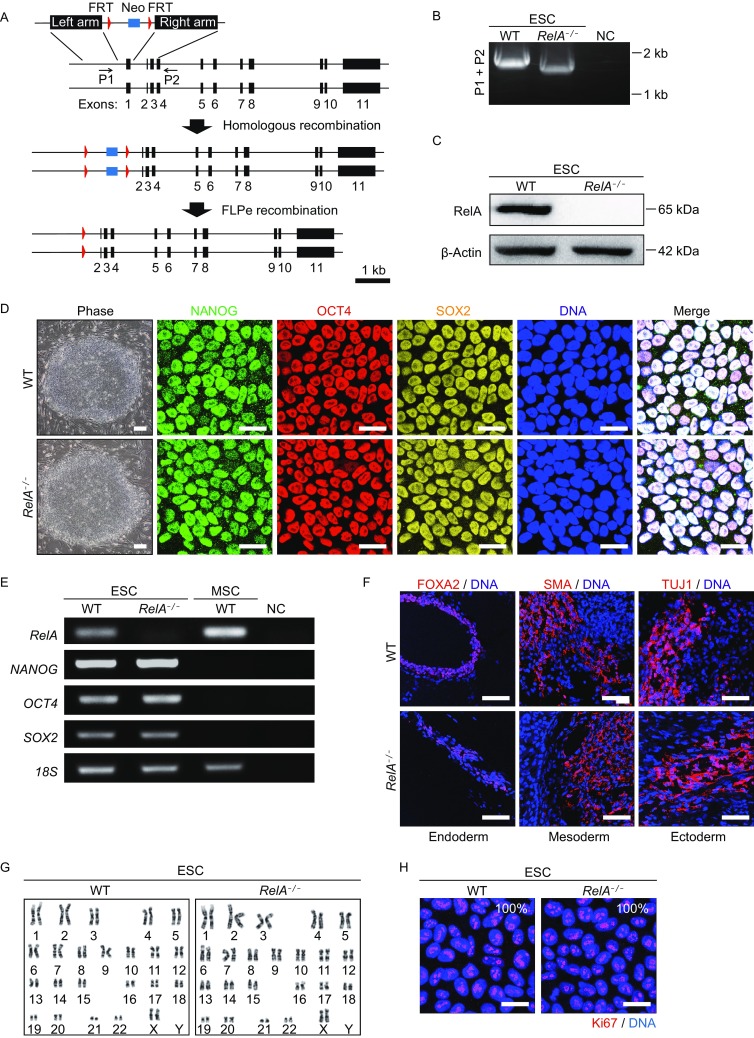
**Generation and characterization of** ***RelA***^***−/−***^ **human ESCs**. (A) Schemic diagram of *RelA* knockout strategy via CRISPR/Cas9 in human ESCs. A neomycin-resistant cassette (Neo) was included for positive selection. (B) Genomic PCR verification of *RelA* exon 1 knockout in *RelA*^*−/−*^ ESCs. Water was used as a negative control (NC). (C) Western blot analysis of RelA protein levels in WT and *RelA*^*−/−*^ ESCs. β-Actin was used as a loading control. (D) Representative colony morphology and immunostaining of pluripotency markers in WT and *RelA*^*−/−*^ ESCs. Scale bar, 30 μm. (E) Measurement of the mRNA expression levels of pluripotency markers by semi-quantitative PCR in WT and *RelA*^*−/−*^ ESCs. *18S* was used as a loading control. (F) Teratoma analysis of WT and *RelA*^*−/−*^ ESCs with three germ layer markers. Markers were stained in red; DNA was labeled in blue by Hoechst 33342. Scale bar, 100 μm. (G) Karyotype analysis of WT and *RelA*^*−/−*^ ESCs. (H) Ki67 immunostaining in WT and *RelA*^*−/−*^ ESCs. Ki67 was stained in red; DNA was labeled by Hoechst 33342. Scale bar, 30 μm

We generated RelA-deficient human ESCs (hESCs) targeting the exon 1 of *RelA* by CRISPR/Cas9-mediated genome editing (Fig. 1A). Successful removal of the targeted exon was verified by PCR (Fig. 1B) and the resulting loss of RelA protein was verified by Western blot (Fig. 1C). The *RelA*^*−/−*^ ESCs exhibited common pluripotent stem cell features including typical colony morphology, expression of pluripotency markers OCT4, SOX2 and NANOG (Fig. 1D and 1E). The *in vivo* differentiation ability of *RelA*^*−/−*^ ESCs was validated by teratoma formation assay (Fig. 1F). Furthermore, karyotype and cell proliferation were each normal in *RelA*^*−/−*^ ESCs when compared to wildtype (WT) controls (Fig. 1G and 1H). These data suggest that the *RelA*^*−/−*^ ESCs maintained typical hESC features.

### Derivation of different human vascular cells from RelA-deficient hESCs

**Figure 2:**
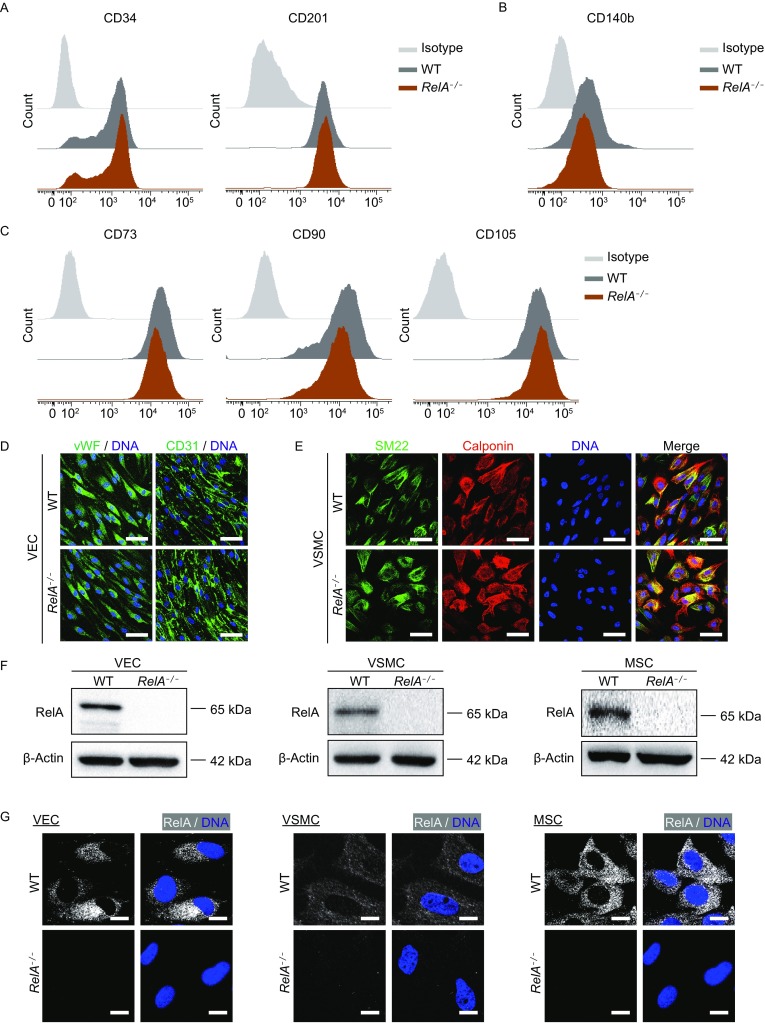
**Derivation of** ***RelA***^***−/−***^ **VECs, VSMCs, and MSCs from** ***RelA***^***−/−***^ **ESCs**. (A) Flow cytometric analysis of WT and *RelA*^*−/−*^ VECs with VEC-specific markers CD34 and CD201. IgG-FITC and IgG-PE were used as isotype controls. (B) Flow cytometric analysis of WT and *RelA*^*−/−*^ VSMCs with VSMC-specific marker, CD140b. IgG-APC was used as an isotype control. (C) Flow cytometric analysis of WT and *RelA*^*−/−*^ MSCs with MSC-specific markers, CD73, CD90 and CD105. IgG-FITC, IgG-PE and IgG-APC were used as isotype controls. (D) Immunostaining of WT and *RelA*^*−/−*^ VECs with VEC-specific markers, vWF and CD31. DNA was labeled by Hoechst 33342. Scale bar, 30 μm. (E) Immunostaining of WT and *RelA*^*−/−*^ VSMCs with VSMC-specific markers, SM22 and Calponin. DNA was labeled by Hoechst 33342. Scale bar, 30 μm. (F) Western blot analysis of RelA protein in WT and *RelA*^*−/−*^ VECs, VSMCs and MSCs, respectively. β-Actin was used as a loading control. (G) Immunostaining of RelA in WT and *RelA*^*−/−*^ VECs, VSMCs and MSCs under basal condition. DNA was labeled by Hoechst 33342. Scale bar, 10 μm

To study how RelA is involved in human vasculature homeostasis, we generated human VECs, VSMCs and MSCs via directed differentiation of *RelA*^*−/−*^ and WT ESCs. Cells were purified by fluorescent-activated cell sorting (FACS) using proper cell surface markers (Fig. 2A–C). Cell purity was confirmed by immunofluorescent staining of additional VEC-specific markers, vWF and CD31 (Fig. 2D) and VSMC-specific markers, SM22 and Calponin (Fig. 2E). While RelA was predominantly retained in the cytoplasm of wildtype vascular cells, loss of RelA protein was verified in different types of RelA-deficient vascular cells by western blotting and immunofluorescent staining (Fig. 2F and 2G).

### RelA deficiency impaired vasculogenesis in VECs and perturbed differentiation potential in MSCs

**Figure 3:**
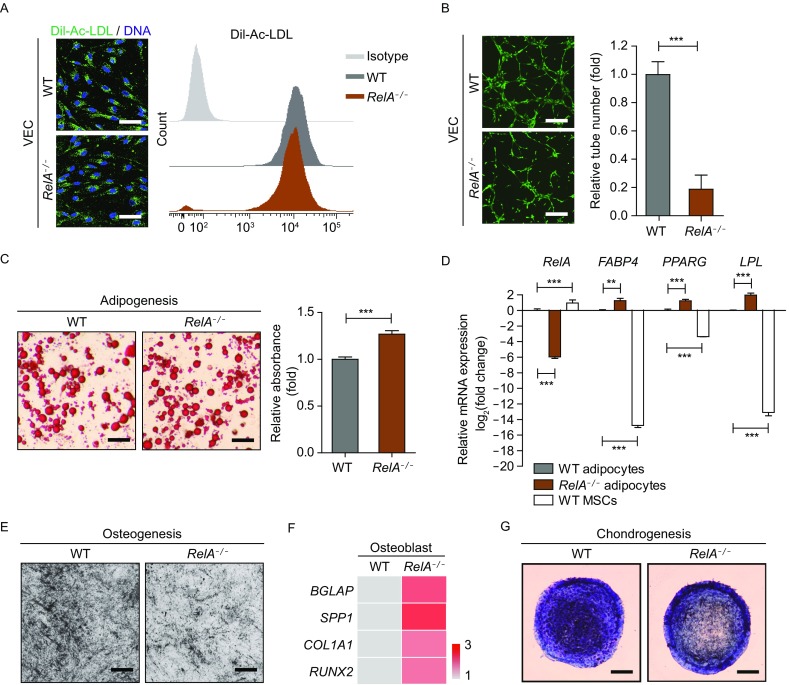
**RelA deficiency affected vascular cell homeostasis**. (A) Immunostaining and flow cytometry analysis of the Dil-Ac-LDL uptake capacity in WT and *RelA*^*−/−*^ VECs. DNA was labeled by Hoechst 33342. Scale bar, 30 μm. (B) Representative micrographs of matrigel tubes formed by WT and *RelA*^*−/−*^ VECs *in vitro* (*n* = 3). Scale bar, 3 mm. (C) Oil red O staining of WT and *RelA*^*−/−*^ adipocytes derived from MSCs, respectively. The quantification of adipocytes was measured by absorbance at 510 nm (*n* = 4). *** *P* < 0.001. Scale bar, 3 mm. (D) Transcriptional expression of adipocyte-specific genes in WT and *RelA*^*−/−*^ adipocytes via RT-qPCR detection (*n* = 4). WT MSCs were used as a negative control. *18S* was used as a loading control. * *P* < 0.05. ** *P* < 0.01. *** *P* < 0.001. (E) Representative micrographs of WT and *RelA*^*−/−*^ osteoblasts by Von Kossa staining. Scale bar, 3 mm. (F) Transcriptional levels of osteoblast-specific gene expression in WT and *RelA*^*−/−*^ osteoblasts via RT-qPCR detection (*n* = 4). *18S* was used as a loading control. (G) Representative toluidine blue staining images of WT and *RelA*^*−/−*^ chondrocytes. Scale bar, 3 mm

We next investigated the functional consequences of RelA deficiency in different vascular cells. Although *RelA*^*−/−*^ VECs had comparable ability to uptake acetylated low-density lipoprotein (Ac-LDL) compared to that of WT VECs (Fig. 3A), RelA deficiency severely interrupted tube formation of VECs *in vitro* (Fig. 3B), indicative of dysregulated VEC function.

Functional MSCs undergo adipogenesis, osteogenesis and chondrogenesis for regeneration *in vivo* (Uccelli et al., 2008). Here we tested whether RelA deficiency interferes with the differentiation potential of MSCs into adipocytes, osteoblasts and chondrocytes. Adipogenesis was slightly enhanced from *RelA*^*−/−*^ MSCs, evidenced by an increase in oil red O staining (Fig. 3C) and upregulation of adipocyte-specific genes like *FABP4*, *PPARG* and *LPL*, which were dramatically enriched in WT adipocytes relative to WT MSCs (Fig. 3D). Despite of increased osteoblast-specific gene expression, there were less calcium deposits stained by Von Kossa in derived *RelA*^*−/−*^ osteoblasts (Fig. 3E and 3F), indicative of aberrant osteogenesis from *RelA*^*−/−*^ MSCs. Moreover, RelA deficiency resulted in defective chondrogenesis with less condensate structure stained by toluidine blue dye (Fig. 3G). These data indicate that RelA was required for maintaining the homeostasis of VECs and MSCs.

### RelA deficiency impeded inflammatory response in human vascular cells

**Figure 4:**
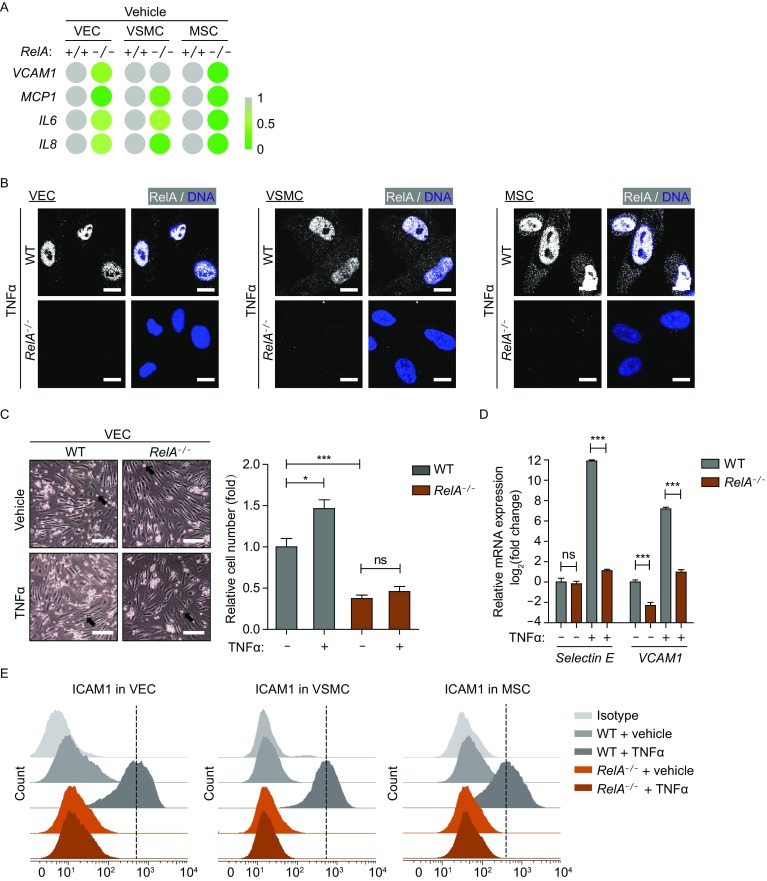
**Inflammatory response defect in** ***RelA***^***−/−***^ **vascular cells**. (A) Transcriptional expression of NF-κB target genes in WT and *RelA*^*−/−*^ VECs, VSMCs, and MSCs under basal condition via RT-qPCR detection (*n* = 4). (B) Immunostaining of RelA in WT and *RelA*^*−/−*^ VECs, VSMCs and MSCs upon 10 ng/mL TNFα treatment. Scale bar, 10 μm. (C) Monocyte adhesion on WT and *RelA*^*−/−*^ endothelium under basal and 10 ng/mL TNFα-induced inflammatory conditions (*n* = 3). Black arrows indicate monocytes. Random fields were selected and the numbers of monocytes were counted by ImageJ software. ns, not significant; * *P* < 0.05; *** *P* < 0.001. (D) Transcriptional expression of adhesion molecules in WT and *RelA*^*−/−*^ VECs under basal and 10 ng/mL TNFα-induced inflammatory conditions via RT-qPCR detection (*n* = 4). ns, not significant. *** *P* < 0.001. (E) Flow cytometric analysis of ICAM1 in WT and *RelA*^*−/−*^ VECs, VSMCs and MSCs under basal and 10 ng/mL TNFα-induced inflammatory conditions. The black dotted line represents the mean ICAM1 protein level upon TNFα treatment. IgG-PE was used as an isotype control

RelA is a well-known component of NF-κB heterodimer, which is a major mediator to regulate inflammation (Barnes and Karin, 1997; Salminen et al., 2008). Accordingly, we measured the mRNA levels of several NF-κB target genes involved in inflammation, including vascular cell adhesion molecule 1 (*VCAM1*), monocyte chemoattractant protein 1 (*MCP1*), *IL6*, and *IL8* in human VECs, VSMCs, and MSCs. Loss of RelA led to reduced mRNA levels of these NF-κB target genes in rested VECs, VSMCs and MSCs (Fig. 4A). As expected, while RelA was translocated from cytosol to nucleus in WT VECs, VSMCs and MSCs upon TNFα treatment, no RelA immunofluorescence signal was observed in *RelA*^*−/−*^ cells (Fig. 4B).

Given that monocyte recruitment to sites of vessel injury and subsequent adhesion to endothelium is critical for inflammation to repair damage and maintain blood vessel homeostasis (Kirton and Xu, 2010; Schober and Weber, 2005), we tested how RelA deficiency affected the adherence of monocytes to endothelium. *RelA*^*−/−*^ VECs exhibited compromised ability in recruiting monocytes compared to WT VECs under basal condition, and this defect was even evident upon TNFα stimulation (Fig. 4C). Furthermore, RelA deficiency resulted in reduced expression of genes implicated in monocyte adhesion to vascular endothelium including *Selectin E*, *VCAM1* and intercellular adhesion molecule 1 (*ICAM1*) upon TNFα treatment (Fig. 4D and 4E). Thus, our data demonstrate that RelA deficiency impeded inflammatory response in human vascular cells.

### RelA deficiency altered proliferative ability in human vascular cells

**Figure 5:**
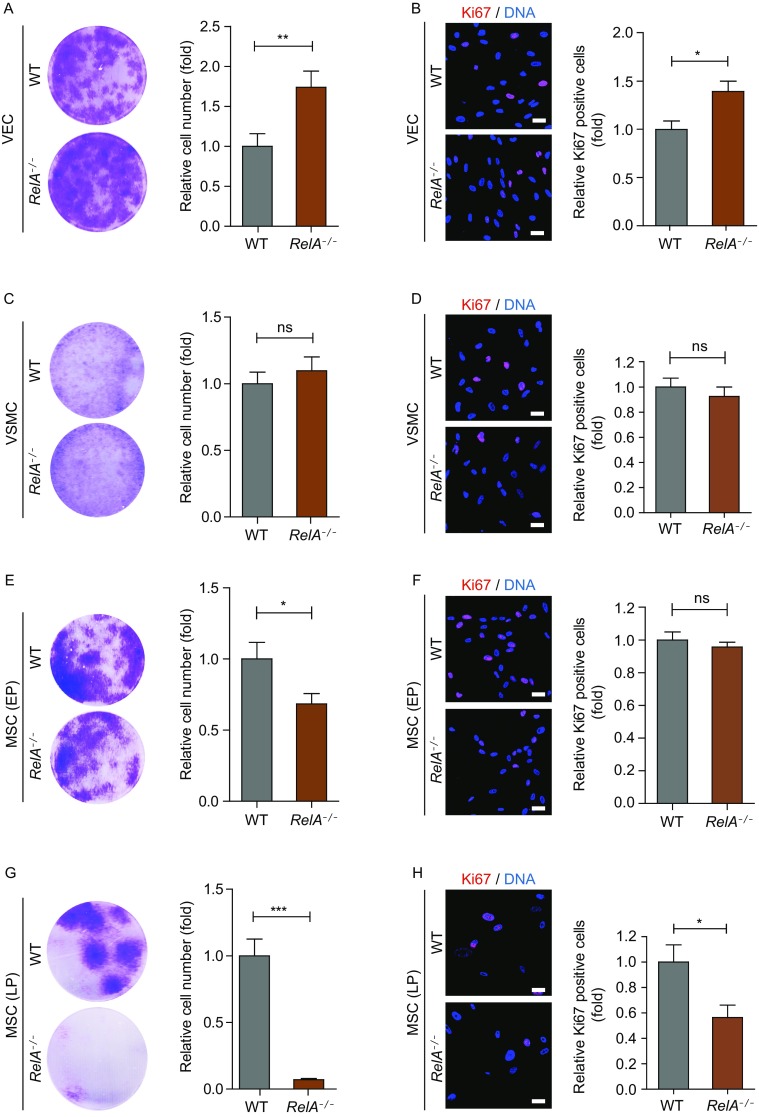
**RelA deficiency resulted in distinct proliferative activity in various vascular cells**. (A) Colony formation of WT and *RelA*^*−/−*^ VECs (*n* = 3). ** *P* < 0.01. (B) Ki67 immunostaining of WT and *RelA*^*−/−*^ VECs (*n* = 3). DNA was labeled by Hoechst 33342. * *P* < 0.05. Scale bar, 30 μm. (C) Colony formation of WT and *RelA*^*−/−*^ VSMCs (*n* = 3). ns, not significant. (D) Ki67 immunostaining of WT and *RelA*^*−/−*^ VSMCs (*n* = 3). DNA was labeled by Hoechst 33342. ns, not significant. Scale bar, 30 μm. (E) Colony formation of WT and *RelA*^*−/−*^ MSCs at passage 3 (early passage, EP) (*n* = 3). * *P* < 0.05. (F) Ki67 immunostaining of WT and *RelA*^*−/−*^ MSCs at passage 3 (early passage, EP) (*n* = 3). DNA was labeled by Hoechst 33342. ns, not significant. Scale bar, 30 μm. (G) Colony formation of WT and *RelA*^*−/−*^ MSCs at passage 7 (late passage, LP) (*n* = 3). *** *P* < 0.001. (H) Ki67 immunostaining of WT and *RelA*^*−/−*^ MSCs at passage 7 (late passage, LP) (*n* = 3). DNA was labeled by Hoechst 33342. * *P* < 0.05. Scale bar, 30 μm

We next checked how RelA affected the proliferative ability of various human vascular cells. RelA deficiency enhanced proliferation in VECs, but not in VSMCs (Fig. 5A–D). In MSCs, however, RelA deficiency led to decreased proliferative ability at early passages (Passage 3) (Fig. 5E and 5F). Given that MSCs as adult stem cells have the self-renewal ability, we further studied how RelA affected MSC proliferation after serial passaging and found even worse proliferative ability in late-passage *RelA*^*−/−*^ MSCs (Passage 7) (Fig. 5G and 5H). Therefore, RelA demonstrated distinct regulatory activities towards the proliferation of VECs, VSMCs and MSCs.

### RelA deficiency promoted TNFα-induced apoptosis in human vascular cells

**Figure 6:**
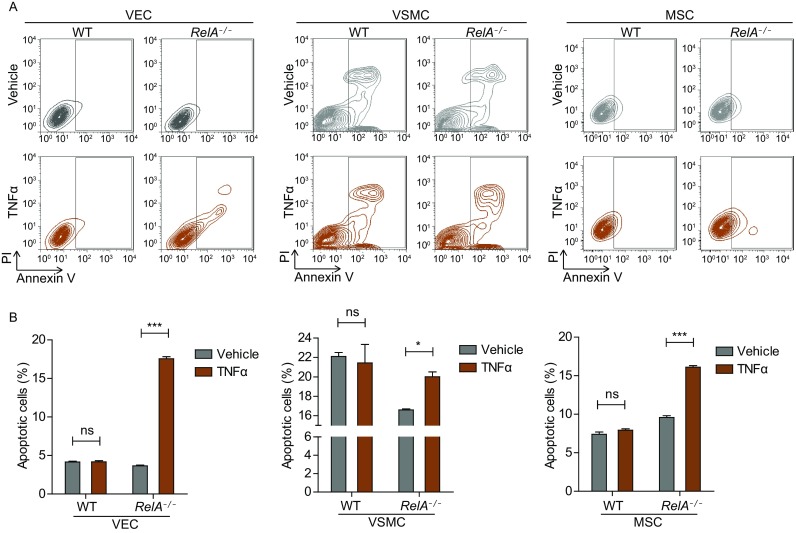
**RelA deficiency promoted TNFα-induced apoptosis**. (A) Flow cytometric analysis of apoptotic vascular cells under basal and 10 ng/mL TNFα-induced conditions. (B) Statistical analysis of apoptotic cells in WT and *RelA*^*−/−*^ VECs, VSMCs, and MSCs upon 10 ng/mL TNFα treatment (*n* = 3). ns, not significant; * *P* < 0.05; *** *P* < 0.001

NF-κB modulates the expression of anti-apoptotic genes, thus promoting cell survival (Kucharczak et al., 2003). Here we tested how RelA affected the survival of human vascular cells under basal and inflammatory conditions. Consistently, the protective effects of NF-κB on inhibiting TNFα-induced apoptosis were impaired by RelA deficiency in various vascular cells. Interestingly, at baseline RelA deficiency exhibited an anti-apoptotic effect in VSMCs, but not in VECs or MSCs (Fig. 6A and 6B). These data indicate that RelA protected human vascular cells against TNFα-induced cell apoptosis.

### MSCs exhibited similar transcriptional signatures upon TNFα or IL1β stimulation

**Figure 7:**
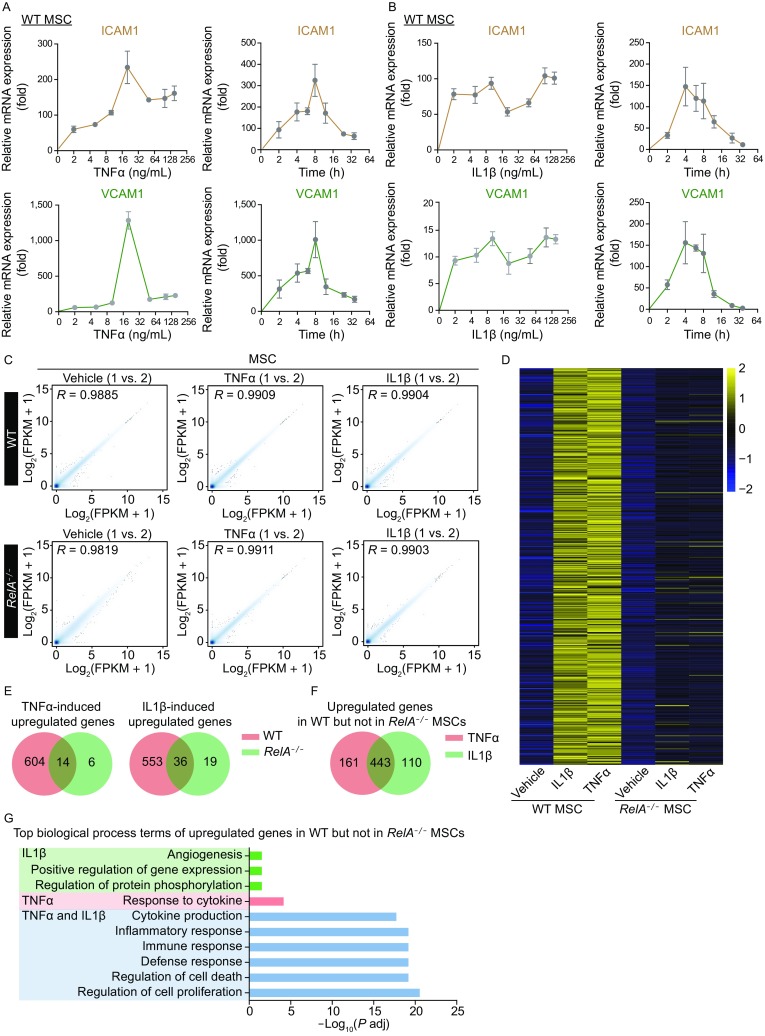
**MSCs exhibited similar transcriptional landscapes upon TNFα or IL1β stimulation**. (A) Transcriptional expression of *ICAM1* and *VCAM1* treated with TNFα at indicated concentrations for 1 h or indicated duration at 10 ng/mL via RT-qPCR detection (*n* = 4). (B) Transcriptional expression of *ICAM1* and *VCAM1* treated with IL1β at indicated concentrations for 1 h or indicated duration at 10 ng/mL via RT-qPCR detection (*n* = 4). (C) Scatter plot showing the correlation between RNA-seq replicates of WT and *RelA*^*−/−*^ MSCs under basal, TNFα (10 ng/mL)- or IL1β (5 ng/mL)-treated conditions. Pearson correlation coefficient (R) is presented. (D) Heatmap showing the Z-score normalized expression levels (FPKM) of coordinately upregulated genes in WT but not in *RelA*^*−/−*^ MSCs upon TNFα or IL1β treatment. (E) Venn diagrams showing the overlap of upregulated genes in WT and *RelA*^*−/−*^ MSCs upon TNFα (left) or IL1β (right) treatment. (F) Venn diagram showing the overlap of upregulated genes in WT but not in *RelA*^*−/−*^ MSCs upon TNFα or IL1β treatment. (G) Gene ontology (GO) enrichment analysis of upregulated genes presented in biological processes affected by the upregulated genes only in IL1β-treated MSCs (green bars), only in TNFα-treated MSCs (red bars), and in both IL1β- and TNFα-treated MSCs (blue bars)

Inflammatory cytokines such as TNFα and IL1β are strong activators of NF-κB signaling pathway (Kida et al., 2005; Osborn et al., 1989). To explore how these cytokines modulate human vascular cells via NF-κB signaling pathway, we measured the mRNA levels of two NF-κB target genes, *ICAM1* and *VCAM1*, upon TNFα or IL1β stimulation in a dose- and time-dependent manner. Upon TNFα induction, the mRNA levels of *ICAM1* and *VCAM1* were both increased with peak expression at 20 ng/mL and by 8 h of treatment (Fig. 7A). By comparison, *ICAM1* and *VCAM1* were upregulated even at low IL1β concentrations with peak expression by 4 h of treatment (Fig. 7B).

We next mapped the transcriptomic landscapes induced by TNFα or IL1β in WT and RelA-deficient MSCs by genome-wide RNA-seq (Fig. 7C). The changes in gene expression patterns were similar upon TNFα or IL1β stimulation in WT MSCs, and were abolished by RelA deficiency (Fig. 7D). A total of 618 upregulated genes induced by TNFα and 589 upregulated genes induced by IL1β were found in WT MSCs, while only 20 upregulated genes induced by TNFα and 55 upregulated genes induced by IL1β were found in *RelA*^*−/−*^ MSCs (Fig. 7E). Most were shared in common (Fig. 7F) and contributed to cell proliferation, survival, defense response, immune response, and inflammatory response (Fig. 7G).

Due to the aforementioned similarity in gene expression patterns upon TNFα or IL1β induction, we used TNFα as the vascular inflammatory stimulus in the subsequent studies.

### Transcriptomic analysis revealed a guardian role of RelA in maintaining blood vessel homeostasis

**Figure 8:**
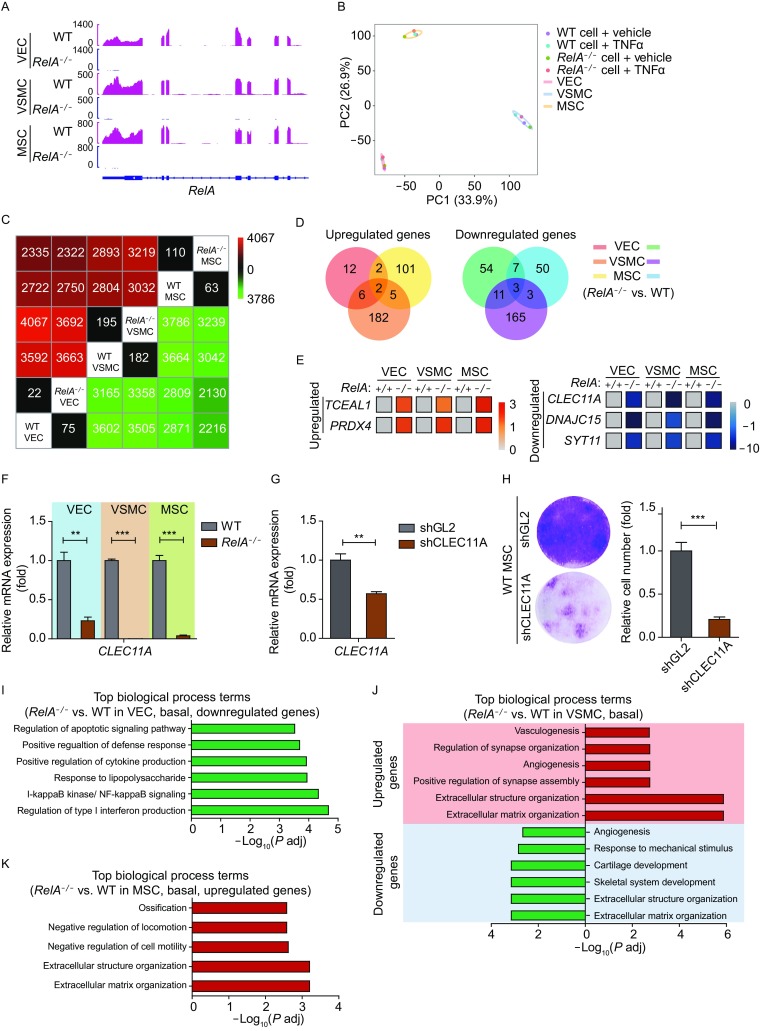
**Transcriptomic analysis revealed RelA deficiency-induced vascular cell dysfunction under basal condition**. (A) Transcriptional signal of RelA in WT and *RelA*^*−/−*^ VECs, VSMCs, and MSCs. Data were normalized by RPKM (reads per kilo bases per million mapped reads) at bin size of 10 bp. (B) Principle component analysis (PCA) of various vascular cells showing a cell type-specific transcriptional pattern. (C) Heatmap showing the number of differential expressed genes between WT and *RelA*^*−/−*^ VECs, VSMCs and MSCs under basal condition. Green color represents downregulated genes; red color represents upregulated genes. (D) Venn diagrams showing the overlap of upregulated (left) and downregulated (right) genes in *RelA*^*−/−*^ VECs, VSMCs and MSCs compared to WT. (E) Heatmap showing the transcriptional levels of upregulated genes (*RelA*^-/-^ vs. WT > 1.5, *P* adj < 0.05) and downregulated genes (*RelA*^-/-^ vs. WT < 0.67, *P* adj < 0.05) in *RelA*^*−/−*^ VECs, VSMCs and MSCs compared to WT. All FPKMs of the genes were normalized to WT groups and the relative gene expression levels are presented via Log_1.5_(*RelA*^-/-^ / WT) in *RelA*^-/-^ groups. (F) Transcriptional expression of *CLEC11A* in WT and *RelA*^*−/−*^ VECs, VSMCs, and MSCs via RT-qPCR detection (*n* = 4). *18S* was used as a loading control. *** *P* < 0.001. (G) Transcriptional expression of *CLEC11A* in WT MSCs infected with shCLEC11A lentiviruses via RT-qPCR detection (*n* = 4). shGL2 lentiviruses were used as a negative control; *18S* was used as a loading control. ** *P* < 0.01. (H) Colony formation of WT MSCs infected with shCLEC11A (*n* = 3). *** *P* < 0.001. (I) GO enrichment analysis of differentially expressed genes in *RelA*^*−/−*^ VECs compared to WT under basal condition. Enriched top GO biological process terms are presented with bars. Green bars represent downregulated genes. (J) GO enrichment analysis of differentially expressed genes in *RelA*^*−/−*^ VSMCs compared to WT under basal condition. Red bars represent upregulated genes; green bars represent downregulated genes. (K) GO enrichment analysis of differentially expressed genes in *RelA*^*−/−*^ MSCs compared to WT under basal condition. Red bars represent upregulated genes

Genome-wide RNA-seq was performed in WT and *RelA*^*−/−*^ VECs, VSMCs and MSCs under basal and TNFα-induced conditions (Fig. S1A and 8A). Under basal condition, RelA deficiency largely retained the transcriptional profile in vascular cells (Fig. 8B and 8C). Venn diagram analysis revealed only two commonly upregulated genes and three downregulated by RelA deficiency among VECs, VSMCs and MSCs (Fig. 8D), despite that RelA expressed substantially in all these cell types (Fig. S1B). These results suggest that RelA modulated vascular cells in a cell type-specific manner. The two commonly upregulated genes were transcription elongation factor A like 1 (*TCEAL1*) and peroxiredoxin 4 (*PRDX4*), and three downregulated genes were C-type lectin domain containing 11A (*CLEC11A*), DnaJ heat shock protein family member C15 (*DNAJC15*), and synaptotagmin 11 (*SYT11*) (Fig. 8E). The expression of *CLEC11A*, one of the downregulated genes and a growth factor that modulates stem cell proliferation and osteogenic development (Hiraoka et al., 2001; Yue et al., 2016), was re-confirmed by qPCR (Fig. 8F). Similarly to our observation with RelA deficiency, *CLEC11A* knockdown also impaired MSC proliferation (Fig. 8G, 8H and S1C), suggesting that RelA deficiency blocked MSC self-renewal at least in part through transcriptional silence of *CLEC11A*.

Further analysis revealed the specific aspects of biological process affected by RelA deficiency. In VECs, RelA deficiency repressed inflammatory response, cytokine production, and NF-κB signaling pathway, supporting a role of RelA in mediating vascular inflammatory response (Fig. 8I). In both VSMCs and MSCs, RelA deficiency led to aberrant extracellular matrix organization and skeletal development that were important to the maintenance of vascular structure and function. In addition, RelA deficiency affected blood vessel formation in VSMCs and promoted the ossification and inhibited the migration of MSCs (Fig. 8J and 8K).

### TNFα-NF-κB axis modulated vascular gene expression in a cell type-specific manner

**Figure 9:**
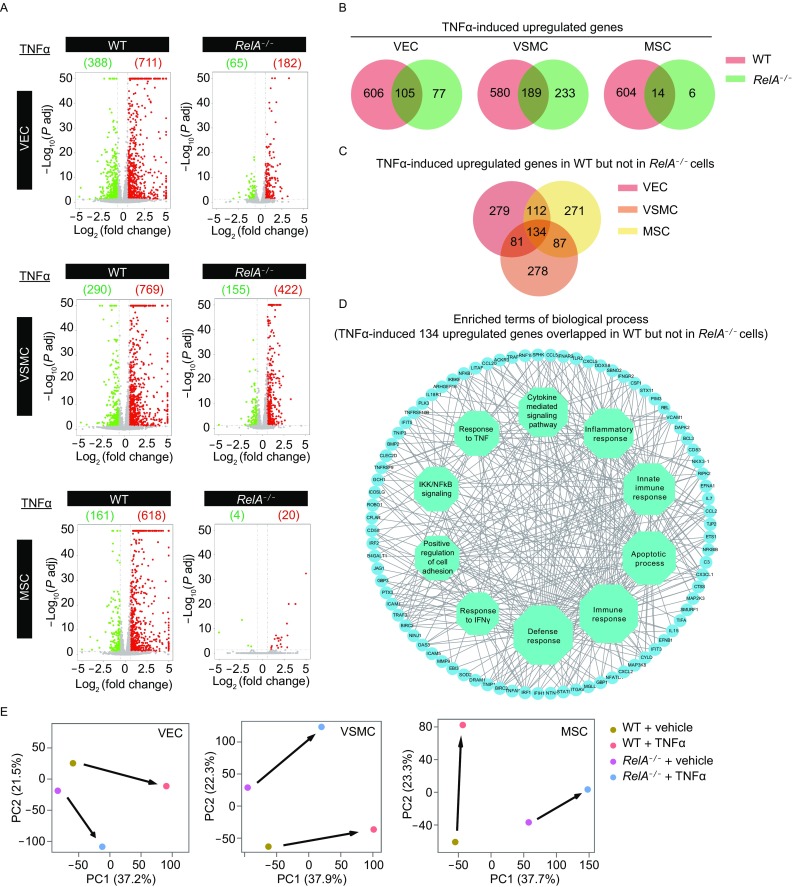
**Transcriptomic analysis revealed RelA deficiency-induced vascular cell dysfunction upon TNFα treatment**. (A) Volcano plots showing the differentially expressed genes between basal and TNFα-treated conditions in WT and *RelA*^*−/−*^ VECs, VSMCs and MSCs. Green color indicates downregulated genes; red color indicates upregulated genes. The number listed in red or green indicates the number of differentially expressed genes. (B) Venn diagrams showing the overlap of upregulated genes in WT and *RelA*^*−/−*^ VECs, VSMCs and MSCs upon TNFα induction. (C) Venn diagram showing the overlap of upregulated genes in WT but not in *RelA*^*−/−*^ VECs, VSMCs, and MSCs upon TNFα induction. (D) Network diagram showing enriched GO biological process terms of upregulated genes in WT but not in *RelA*^*−/−*^ VECs, VSMCs and MSCs upon TNFα treatment. The size of octagons represents the number of upregulated genes enriched in each term of biological processes. (E) Principal component analysis (PCA) showing a transcriptional pattern shift upon TNFα induction in WT and *RelA*^*−/−*^ VECs, VSMCs and MSCs

We next investigated how RelA regulated gene expression in TNFα-treated vascular cells. RelA deficiency dramatically abrogated TNFα-induced transcriptional changes in all types of vascular cells, especially in MSCs (Fig. 9A). Venn diagram analysis revealed that a total of 606 genes in WT VECs, 580 genes in WT VSMCs, and 604 genes in WT MSCs were upregulated by TNFα in a RelA-dependent manner (Fig. 9B). In VECs, TNFα induced a set of genes that modulate inflammatory response and cytokine signaling pathway (Fig. S1D). In VSMCs the upregulated genes contributed to defense response and cell proliferation (Fig. S1E). In MSCs the induced genes were associated with immune response and apoptotic process (Fig. S1F). All these effects were abolished in *RelA*^*−/−*^ vascular cells.

Next, we compared the three sets of TNFα-induced RelA-target genes in WT VECs, VSMCs and MSCs. A total of 134 genes were commonly upregulated among the three types of vascular cells (Fig. 9C). These genes contributed to biological processes including immune response, cytokine signaling, cellular adhesion and apoptotic process, indicating that they may be critical to protect vascular cells from inflammatory damage in a NF-κB-dependent manner (Fig. 9D and S1G). Furthermore, PCA analysis revealed that RelA deficiency increased the transcriptomic differences in vascular cells upon TNFα stimulation (Fig. 9E).

Taken together, our data demonstrate that RelA deficiency interfered with inflammatory response and cytokine signaling in vascular cells, indicating a protective role of RelA in maintaining vascular homeostasis during vascular inflammation.

### Effect of IκBα deficiency on NF-κB pathway

**Figure 10:**
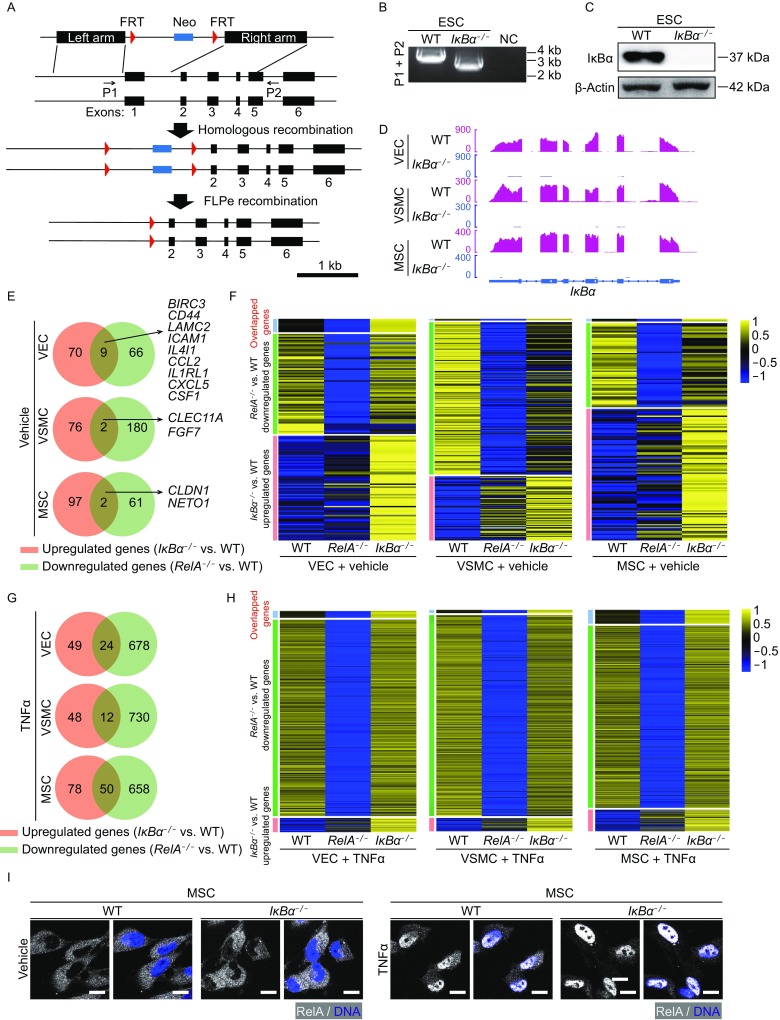
**Transcriptomic analysis revealed the effect of IκBα deficiency on RelA signaling**. (A) Schemic diagram of *IκB*α knockout strategy via CRISPR/Cas9 in human ESCs. A neomycin-resistant cassette (Neo) was included for positive selection. (B) Genomic PCR verification of the deletion of *IκB*α exon 1 in *IκB*α^*−/−*^ ESCs. Water was used as a negative control (NC). (C) Western blot analysis showing IκBα protein levels in WT and *IκB*α^*−/−*^ ESCs. β-Actin was used as a loading control. (D) Transcriptional signal of IκBα in WT and *IκB*α^*−/−*^ in VECs, VSMCs and MSCs. Transcriptional signals were normalized by RPKM at bin size 10 bp. (E) Venn diagrams showing the overlap between upregulated genes in *IκB*α^*−/−*^ vascular cells and downregulated genes in *RelA*^*−/−*^ vascular cells compared to WT vascular cells under basal condition. (F) Heatmaps revealing the transcriptional patterns of genes upregulated only in *IκB*α^*−/−*^ vascular cells (pink), downregulated only in *RelA*^*−/−*^ vascular cells (green), and genes overlapped (blue) under basal condition. (G) Venn diagrams showing the overalp between upregulated genes in *IκB*α^*−/−*^ vascular cells and downregulated genes in *RelA*^*−/−*^ vascular cells compared to WT vascular cells upon TNFα treatment. (H) Heatmaps revealing the transcriptional patterns of genes upregulated only in *IκB*α^*−/−*^ vascular cells (pink), downregulated only in *RelA*^*−/−*^ vascular cells (green) and genes overlapped (blue) upon TNFα treatment. (I) Immunostaining of RelA in WT and *IκB*α^*−/−*^ MSCs under basal and TNFα-treated conditions. DNA was labeled by Hoechst 33342. Scale bar, 10 μm

Previous studies have shown that IκBs are essential for the inhibition of RelA and that RelA can be released and activated under inflammatory stimulus. To investigate whether NF-κB is negatively modulated by IκBs in vascular cells, we generated *IκB*α knockout (*IκB*α^*−/−*^) hESCs by targeting the first exon of *IκB*α using CRISPR/Cas9-mediated genome editing (Fig. 10A). Removal of the targeted exon was verified in *IκB*α^*−/−*^ ESCs (Fig. 10B) and the resulting protein loss was confirmed by western blotting (Fig. 10C). To further investigate the role of IκBα in vascular cells, we differentiated WT and *IκB*α^*−/−*^ ESCs to VECs, VSMCs and MSCs, respectively. Loss of IκBα expression was verified in diverse *IκB*α^*−/−*^ cells (Fig. 10D).

To understand how IκBα regulates the homeostasis of different vascular cells, we performed genome-wide RNA-seq in WT and *IκB*α^*−/−*^ VECs, VSMCs, and MSCs under basal and TNFα-induced conditions (Fig. S2A). IκBα deficiency resulted in 79, 78 and 99 genes upregulated, and 104, 97, 90 genes downregulated in untreated VECs, VSMCs, and MSCs, respectively (Fig. S2B). Venn diagram and GO enrichment analysis revealed that IκBα regulated gene expression in a cell type-specific manner (Fig. S2B–E). To further determine whether RelA modulates gene expression dependently of IκBα, we compared the upregulated genes in *IκB*α^*−/−*^ vascular cells with the downregulated genes in *RelA*^*−/−*^ vascular cells. Only a small fraction of genes were overlapped in each of the three vascular cell types (Fig. 10E–H), regardless of the absence or presence of TNFα. Consistently, we did not observe marked differences in the subcellular localization of RelA in IκBα-depleted cells (Fig. 10I). These data suggest that IκBα acted largely independent of RelA in various human vascular cells.

## Discussion

**Figure 11:**
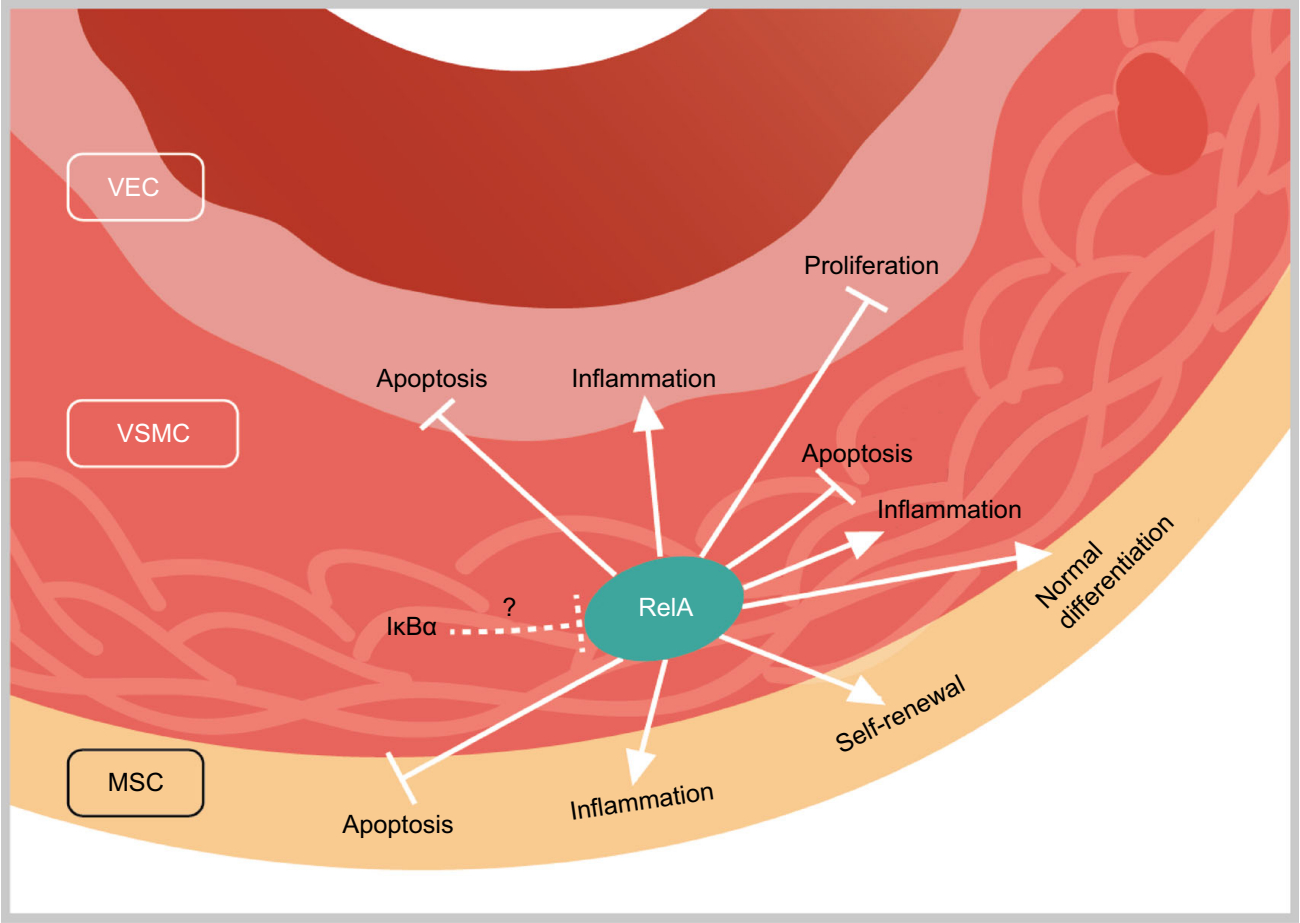
**A proposed model showing a guardian role of RelA in maintaining human vascular cell homeostasis**. (A) Under basal condition, NF-κB/RelA inhibits the proliferation of vascular endothelial cells (VECs) and promotes the self-renewal of mesenchymal stem cells (MSCs) that are the crucial cell type of the adventitia layer. NF-κB/RelA also maintains the multi-differentiation potentials of MSCs. Upon TNFα treatment, RelA mediates vascular inflammation and protects vascular cells from apoptosis. This model suggests a guardian role of RelA in maintaining human blood vessel homeostasis. Notably, IκBα appears to modulate vascular gene expression predominantly in a RelA-independent manner under basal and inflammatory conditions

In this study, we generated RelA-deficient human ESCs using CRISPR/Cas9-mediated genome editing and differentiated them into different types of vascular cells to investigate how NF-κB/RelA regulates human vascular cells under basal and inflammatory conditions. Via genome-wide RNA sequencing analysis, we mapped the NF-κB-regulated transcriptomic landscapes to systematically understand the physiological roles of RelA in various vascular cells. Our findings suggest that RelA modulated vascular cells in a cell type-specific manner by affecting multiple aspects of cellular events including extracellular matrix organization, ossification, vasculogenesis, inflammatory response, proliferation and survival. In addition, our data indicate that IκBα regulated gene expression in vascular cells primarily in a RelA-independent manner (Fig. 11).

The correlation between NF-κB activation and vascular dysfunction have attracted great attention during the last decades. For instance, the nuclear translocation of RelA has frequently been observed in human atherosclerotic lesion vascular cells (Brand et al., 1996; Mallavia et al., 2013). In *ApoE*^*−/−*^ mice the activation of NF-κB signaling pathway in endothelial cells and smooth muscle cells is accompanied with enhanced secretion of inflammatory factors and increased atherosclerotic lesions (Gareus et al., 2008; Mallavia et al., 2013). In addition, TNFα-induced NF-κB activation impairs cell proliferation and causes premature senescence in HUVECs (Khan et al., 2017); knockdown of RelA promotes cell proliferation and reduces apoptosis in high glucose-treated HUVECs (Chen et al., 2011a). Yet, the role of NF-κB in different human vascular cells has not been well studied via a side-by-side comparison.

Here, we took advantage of the various types of differentiated RelA-deficient vascular cells to reveal the physiological functions of NF-κB in these cells, providing direct, unbiased characterization of the cell type-specific transcriptional regulation by NF-κB in vascular cells. Our platform is of superior advantages over traditional tools such as gene-engineered mice (Ijaz et al., 2016), RNAi-mediated knockdown in human vascular cells (Chen et al., 2011a; Chen et al., 2011b), as well as small-molecule agonists and antagonists of NF-κB (Jakkampudi et al., 2016; Janssen-Heininger et al., 2000).

Our data provided a comprehensive understanding on how NF-κB regulates the homeostasis of various human vascular cells under basal and inflammatory conditions. For instance, we observed enhanced adipogenesis and osteogenesis in *RelA*^*−/−*^ MSCs, which suggests that RelA deficiency may cause atherosclerosis-associated abnormal lipid accumulation and vascular ossification. Our data also showed that the self-renewal and differentiation abilities of MSCs were directly regulated by RelA, implying that RelA inactivation might cause vessel dysfunction via compromising the regenerative ability of the adventitia-localized stem cells. Furthermore, we showed that RelA deficiency impaired vasculogenesis in VECs and VSMCs. Thus, our study offers detailed illustrations how RelA regulates gene expression in a vascular cell type-specific manner and supports the notion that RelA is implicated in the multi-perspective maintenance of human vascular homeostasis.

IκBs sequester NF-κB in the cytoplasm, thus inhibiting NF-κB activity. Degradation of IκBs releases NF-κB, leading to its translocation to the nucleus and the subsequent activation of NF-κB target genes (Simeonidis et al., 1999). There are at least eight dedicated IκB proteins: IκBα, IκBβ, IκBγ, IκBε, IκBz, IκBNS, Bcl-3 and Drosophila Cactus (Morris et al., 2016), with IκBα generally regarded as the predominant isoform (Fagerlund et al., 2015). In patients, heterozygous nonsense mutation in IκBα leads to NF-κB haploinsufficiency, resulting in ectodermal dysplasia and immune deficiency (Courtois et al., 2003). In this study, we genetically ablated IκBα in hESCs and obtained various vascular cells via directed differentiation to study the impact of IκBα deficiency on the functions of NF-κB signaling. Our results showed that IκBα acted largely independent of RelA signaling under basal condition as well as upon TNFα stimulation, suggesting that NF-κB is not predominantly regulated by IκBα, but perhaps by other IκB isoforms in vascular cells.

To date, inhibition of NF-κB during vascular inflammation has provided a promising therapeutic target for the treatment of various cardiovascular disorders. Various inhibitors that target NF-κB pathway have been developed to extend the choice for clinical applications (Tas et al., 2009). In our study, we have provided a platform that facilitates the in-depth mechanistic interpretation of how RelA regulates human vascular diseases and aging. The differential responses in various types of vascular cells to RelA deficiency sound a cautionary note for diseases being considered for anti-NF-κB treatment. Moderate suppression is likely to be beneficial to the maintenance of vascular homeostasis by alleviating the injury from vascular inflammation, whereas excessive suppression may lead to the abnormal behaviors for different layers of vascular cells. Lastly, given that the regulation of NF-κB is of cell type specificity, our study has demonstrated the importance of highly targeted therapy in the treatment of vascular inflammatory diseases via the inhibition of NF-κB activity.

## Materials and methods

### Cell culture

H9 human ESCs purchased from WiCell Research institute were cultured on mitomycin C-inactivated mouse embryonic fibroblasts (MEFs) or matrigel (BD Biosciences) (Liu et al., 2012). Human VECs were cultured in EGM-2 (Lonza) medium supplemented with 50 ng/mL VEGF (HumanZyme), 10 μmol/L SB431542 (Selleck) and 20 ng/mL FGF2 (Joint Protein Central, JPC). Human VSMCs were cultured in N2B27 medium (1:1 (*v*/*v*) of DMEM/F12 medium (Gibco) and Neurobasal medium (Gibco) supplemented with 2% B27 (Gibco), 1% N2 (Gibco), 55 μmol/L β-mercaptoethanol (Gibco) and 1% penicillin/streptomycin (Gibco)) supplemented with 10 ng/mL PDGF-BB (Peprotech). Human MSCs were cultured in 90% α-MEM medium (Gibco) supplemented with 10% fetal bovine serum (Ausbian, VS500T), 1% penicillin/streptomycin (Gibco), and 1 ng/mL FGF2 (JPC).

### Generation of *RelA*^*−/−*^ ESCs and *IκB*α^*−/−*^ ESCs

CRISPR/Cas9 mediated gene targeting was performed as previously described (Ding et al., 2013; Wang et al., 2017; Wang et al., 2018). In brief, a donor plasmid containing homologous arms was constructed with a neomycin resistant cassette. sgRNAs targeting *RelA* (sequence: CTC GTC TGT AGT GCA CGC CGC GG) and *IκB*α (sequence: GCT CCT TCT TCA GCC CGT CG) were also constructed in the plasmid respectively. 5 × 10^6^ ESCs were digested by TrypLE and were electroporated with 7 μg Cas9 plasmid, 7 μg *RelA* or *IκB*α targeted sgRNA plasmid and 7 μg donor plasmid. Cells were then cultured on DR4 MEF feeder. G418 (Gibco) was used for targeting screening for 14 days, and the drug resistant clones were transported to a 96-well plate for expansion and RelA or IκBα knockout verification. The targeted clones were verified by genomic PCR with primers listed as follows, *RelA* P1: CCA TCC GGG CTG TAG GCT CCG CAA AGC TC, P2: TCA TAG CCC GCC TCC TGT CCC CTC ATG CTG. *IκB*α P1: GCC CAG CCA TCA TTT CCA CTC TTG CGT TT, P2: AAG CAA CAA AAT GAG GGC TGA TCC TAC CAC. The neomycin-resistant cassette was later removed after verification according to the method previously described (Duan et al., 2015).

### VEC generation and characterization

VECs were differentiated as previously described (Pan et al., 2016; Wu et al., 2018). In brief, human ESC clones were seeded in matrigel coated 6-well plates in mTeSR medium at day 0. ESCs were washed with IMDM medium (Gibco) and cultured in EGM-2 medium (Lonza) supplemented with 25 ng/mL BMP4 (R&D), 3 μmol/L CHIR99021 (Selleck), 3 μmol/L IWP2 (Selleck) and 4 ng/mL FGF2 (JPC) for 3 days. At day 4, Cells were rinsed with IMDM medium and cultured in EGM-2 medium supplemented with 50 ng/mL VEGF (HumanZyme), 10 ng/mL IL6 (Peprotech) and 20 ng/mL FGF2 (JPC) for 3 days. Endothelial cells were sorted with CD201-PE (Biolegend, 351904, 1:300), CD34-FITC (BD biosciences, 555821, 1:100). IgG-FITC (BD biosciences, 555748) and IgG-PE (BD biosciences, 555749) were used as isotype controls.

### VSMC generation and characterization

VSMCs were differentiated as previously reported (Patsch et al., 2015). Human ESC clones were digested by Accutase (Gibco) and 3 × 10^5^ cells were seeded in matrigel coated 6-well plate and mTeSR medium containing 10 μmol/L Y27632 (Selleck) at day 0. Cells were cultured in the N2B27 medium with 25 ng/mL BMP4 (R&D), 8 μmol/L CHIR99021 (Selleck) for 3 days. At day 4, cells were cultured in N2B27 medium with 10 ng/mL PDGF-BB (Peprotech), 2 ng/mL Activin A (HumanZyme) and the medium was changed every day for 2 days. VSMCs were sorted with CD140b-APC (BD biosciences, 558821, 1:200), and IgG-APC (BD Biosciences, 555751) was used as an isotype control.

### MSC generation and characterization

MSCs were derived from ESCs according to a protocol previously described (Zhang et al., 2015). Briefly, human ESC clones from embryoid bodies (EBs) were cultured in 90% α-MEM medium (Gibco) with 10% FBS (Ausbian, VS500T), 1% penicillin/streptomycin (Gibco), 5 ng/mL TGFβ (HumanZyme) and 10 ng/mL FGF2 (JPC) for 10 days until fibroblast-like cells emerged. The MSCs were sorted for CD73^+^/CD90^+^/CD105^+^ cells by FACS. Antibodies for sorting and characterization were CD73-PE (BD Biosciences, 550257), CD90-FITC (BD Biosciences, 555595), CD105-APC (eBioscience, 555748). IgG-FITC, IgG-PE, and IgG-APC were used as isotype controls. The differentiation abilities of MSCs to adipocytes, osteoblasts and chondrocytes were assessed by oil red O, Von Kossa and Toluidine blue staining, respectively (Duan et al., 2015; Fang et al., 2018; Yang et al., 2017; Zhang et al., 2015).

### Flow cytometry analysis

Cell apoptosis analysis was performed with Annexin V-EGFP Apoptosis Detection Kit (Vigrous Biotechnology). Cells were pre-treated with 10 ng/mL TNFα (Peprotech) for 24 h before performing apoptosis assay. For ICAM1 expression analysis, cells were suspended in 10% FBS/PBS with ICAM1-PE (BD Biosciences, 560971, 1:100) for 30 min, and IgG-PE was used as an isotype control. The results were determined by FlowJo7.6.1 software. For Dil-Ac-LDL uptake analysis, VECs were cultured with Dil-Ac-LDL (Molecular probes, 1:400) for 6–8 h at 37 °C. VECs were harvested and washed twice by PBS and analyzed by FACS detected by FITC channel.

### Western blotting

Protein was quantified by a BCA Kit after cells were lysed in RIPA buffer as previously described (Duan et al., 2015). 20 μg of protein lysates were subjected to 10% SDS-PAGE and electrotransferred to a 0.22 μm PVDF membrane (Millipore). The membrane was incubated in primary antibody overnight at 4 °C and HRP-conjugated secondary antibody followed by visualization using chemi-luminescence. The antibodies used were as follows: anti-RelA (Abcam, ab7970, 1:1000), anti-IκBα (Santa Cruz, sc-371, 1:1000) and anti-β-Actin (Santa Cruz, sc-69879, 1:4000).

### Immunofluorescence

Cells were fixed in 4% formaldehyde for 30 min, permeabilized in 0.4% Triton X-100 in PBS for 30 min and incubated with blocking buffer (10% donkey serum in PBS) for 30 min. Samples were incubated with primary antibody overnight in 4 °C and with secondary antibody for 1 h at room temperature. Cell images were taken using confocal microscopy. Nuclear DNA was stained by Hoechst 33342 (Invitrogen, H3570). Antibodies used were as follows: anti-NANOG (Abcam, ab21624, 1:200), anti-SOX2 (Santa Cruz, sc-17320, 1:100), anti-OCT4 (Santa Cruz, sc-5279, 1:100), anti-TUJ1 (Sigma, T2200, 1:100), anti-αSMA (Sigma, A5228, 1:100), anti-FOXA2 (Cell Signaling Technology, 8186S, 1:100), anti-CD31-FITC (BD Biosciences, 555445, 1:50), anti-vWF (Dako, A0082, 1:200), Dil-Ac-LDL (Molecular probes, 1:400), anti-SM22 (Abcam, ab14106, 1:200), anti-Calponin (BD Biosciences, 2017-03, 1:200) and anti-Ki67 (Vector Labs, VP-RM04, 1:1,000) anti-RelA (Cell Signaling Technology, 8242, 1:200).

### RNA extraction and RT-qPCR

Cells were harvested in TRIzol reagent (Invitrogen) and RNA was extracted according to the manufacturers’ instruction. 2 μg RNA was used for cDNA synthesis using reverse transcription master Mix (Promega). RT-qPCR was performed in SYBR Green supermix (Thunderbird) on a CFX-384 RT-qPCR system. The relative expression of genes was normalized by *18S* rRNA transcript. All qPCR primers used were listed as follows: *18S* forward primer, GTA ACC CGT TGA ACC CCA TT, reverse primer, CCA TCC AAT CGG TAG TAG CG. *RelA* forward primer, GTG GGG ACT ACG ACC TGA ATG, reverse primer, GGG GCA CGA TTG TCA AAG ATG. *NANOG* forward primer, ACA ACT GGC CGA AGA ATA GCA, reverse primer, GGT TCC CAG TCG GGT TCA C. *OCT4* forward primer, GGG TTT TTG GGA TTA AGT TCT TCA, reverse primer, GCC CCC ACC CTT TGT GTT. *SOX2* forward primer, CAA AAA TGG CCA TGC AGG TT, reverse primer, AGT TGG GAT CGA ACA AAA GCT ATT. *FABP4* forward primer, ACT GGG CCA GGA ATT TGA CG, reverse primer, CTC GTG GAA GTG ACG CCT T. *PPARG* forward primer, ACC AAA GTG CAA TCA AAG TGG A, reverse primer, ATG AGG GAG TTG GAA GGC TCT. *LPL* forward primer, TCA TTC CCG GAG TAG CAG AGT, reverse primer, GGC CAC AAG TTT TGG CAC C. *BGLAP* forward primer, CAC TCC TCG CCC TAT TGG C, reverse primer, CCC TCC TGC TTG GAC ACA AAG. *SPP1* forward primer, CTC CAT TGA CTC GAA CGA CTC, reverse primer, CAG GTC TGC GAA ACT TCT TAG AT. *COL1A1* forward primer, GTG CGA TGA CGT GAT CTG TGA, reverse primer, CGG TGG TTT CTT GGT CGG T. *RUNX2* forward primer, CCG CCT CAG TGA TTT AGG GC, reverse primer, GGG TCT GTA ATC TGA CTC TGT CC. *ICAM1*, forward primer, ATG CCC AGA CAT CTG TGT CC, reverse primer, GGG GTC TCT ATG CCC AAC AA. *VCAM1* forward primer, TTT GAC AGG CTG GAG ATA GAC T, reverse primer, TCA ATG TGT AAT TTA GCT CGG CA. *MCP1* forward primer, CAG CCA GAT GCA ATC AAT GCC, reverse primer, TGG AAT CCT GAA CCC ACT TCT. *IL6* forward primer, ACT CAC CTC TTC AGA ACG AAT TG, reverse primer, CCA TCT TTG GAA GGT TCA GGT TG. *IL8* forward primer, ACT GAG AGT GAT TGA GAG TGG AC, reverse primer, AAC CCT CTG CAC CCA GTT TTC. *Selectin E* forward primer, CAG CAA AGG TAC ACA CAC CTG, reverse primer, CAG ACC CAC ACA TTG TTG ACT T. *CLEC11A* forward primer, GGG CCT CTA CCT CTT CGA AA, reverse primer, CAG TTC TCG AGC GTG CCA CC.

### RNA-seq library construction

Vascular cells were cultured and treated with TNFα at 10 ng/mL for 4 h before harvest. Alternatively, MSCs were treated with IL1β at 5 ng/mL for 4 h before harvest. RNA-seq libraries were constructed following a previous protocol (Li et al., 2016). RNA integrity was verified by the Bioanalyzer 2100 system (Agilent Technologies). Sequencing libraries were obtained using NEBNext^®^ Ultra^TM^ RNA Library Prep Kit for Illumina^®^ (NEB) and index codes were tagged to each samples for sequencing. The libraries were sequenced on an Illumina Hiseq platform.

### RNA-seq data processing

RNA-seq data processing was performed as previously reported (Li et al., 2016). Briefly, low quality reads were trimmed and then paired clean reads were mapped to hg19 human genome using hisat2 (v2.0.4) (Kim et al., 2015). Reads were then counted by HTSeq (v0.6.1) (Anders et al., 2015). Differentially expressed genes (DEGs) were calculated using DESeq2 with the cutoff Benjamini–Hochberg adjust P value 0.05 and absolute fold change more than 1.5 (Love et al., 2014). To evaluate the correlation between replicates of each sample, the Pearson correlation coefficient (R) was computed based on log_2_(FPKM + 1). Gene Ontology (GO) and KEGG pathway enrichment analysis was carried out by ClusterProfiler R package (Yu et al., 2012). The RNA-seq data have been deposited to the NCBI Gene Expression Omnibus (GEO) database with accession number GSE115311.

### Matrigel tube formation assay

2 × 10^4^ VECs were seeded in each well of a 24-well plate coated with matrigel (BD Biosciences) in triplicate and incubated for 6–8 h at 37 °C, and then stained by Calcein-AM (Invitrogen) (Wu et al., 2018). The tube formation was assessed by taking photomicrographs and measured by ImageJ2x 2.1.4.7 software.

### Monocyte adhesion assay

2 × 10^5^ VECs were seeded in each well of 12-well plate coated with collagen. The next day, VECs were treated with or without 10 ng/mL TNFα for 4 h. 2 × 10^6^ monocytes were co-cultured with VECs for 1 h and then rinsed by PBS for 3 times carefully (Lee et al., 2015). The monocytes adhered on endothelium were analyzed by ImageJ2x 2.1.4.7 software.

### Colony formation assay

VECs, VSMCs and MSCs were each seeded by 2,000 cells per well in 12-well plates (Pan et al., 2016), and cultured until clear cell colonies formed. Cells were fixed in 4% paraformaldehyde for 30 min and stained with crystal violet (Biohao Biotechnology). The images were analyzed by ImageJ software.

### ShRNA plasmid construction and lentivirus packaging

ShRNA sequence (TGA GGA CAT CGT CAC TTA C) that targeting CLEC11A was cloned into pLVTHM vector. For lentivirus packaging, CLEC11A shRNA was co-transfected with psPAX2 (Addgene) and pMD2.G (Addgene) into HEK293T cells. Lentiviruses were harvested after 48 h and ultracentrifuged at 19,400 *g* for 2.5 h followed by resuspension in α-MEM medium (Kubben et al., 2016; Liu et al., 2011).

### Teratoma experiment

3 × 10^6^ human ESCs were injected into NOD-SCID (Non-obese diabetic severe combined immunodeficiency) mice under subcutaneous regions. After three months, mice were sacrificed and teratomas were excised (Wu et al., 2018). The teratomas were fixed in 4% paraformaldehyde, dehydrated in 30% sucrose solution, embedded in O.C.T compound and frozen in liquid nitrogen. Samples were sectioned and analyzed by immunofluorescent staining. The animal experiments were approved by the Institute of Biophysics, Chinese Academy of Sciences.

### Statistical analysis

All the results were presented as mean ± SEM. Two-tailed Student`s *t*-test was performed to compare the differences between two groups. All quantitative experiments were repeated at least 3 times independently.

## Electronic supplementary material

Below is the link to the electronic supplementary material.

Supplementary material 1 (PDF 5,810 kb)
