# Supplementary material for: CRISPR/Cas9-mediated gene knockout reveals a guardian role of NF-κB/RelA in maintaining the homeostasis of human vascular cells
Source: Protein Cell. 2018 Jul 2;9(11):945–65. doi: 10.1007/s13238-018-0560-5 (PMC6208479; doi:10.1007/s13238-018-0560-5)
Supplement: Supplementary file 1 — Supplementary material 1 (PDF 5,810 kb) [file 13238_2018_560_MOESM1_ESM.pdf]

Figure S1. Biological processes modulated by NF-κB in various vascular cells under inflammatory conditions.

a) Heatmap showing the pairwise correlation of RNA-seq signals between WT and *RelA*$^{-/-}$ vascular cells under basal condition and upon TNFα treatment. Pearson correlation coefficient (R) is presented to the right.
b) Western blot analysis of RelA protein levels in WT VECs, VSMCs and MSCs under basal condition (n=3). The number below represents the relative expression of RelA in various vascular cells. β-Actin was used as a loading control.
c) Ki67 immunostaining of WT MSCs infected with shCLEC11A viruses. shGL2 viruses were used as a negative control. DNA was labeled by Hoechst 33342. ***p< 0.001. Scale bar, 30 μm.
d) GO enrichment analysis of upregulated genes only in WT VECs upon TNFα treatment. Enriched top 10 GO biological processes are presented with dots. The area of dot represents the count of genes in the terms.
e) GO enrichment analysis of upregulated genes upon TNFα treatment only in WT VSMCs.
f) GO enrichment analysis of upregulated genes upon TNFα treatment only in WT MSCs.
g) GO enrichment analysis of upregulated genes upon TNFα induction among WT but not *RelA*$^{-/-}$ VECs, VSMCs and MSCs.

Figure S2. RNA-seq analysis identified IκBα-regulated genes in vascular cells.

a) Correlation analysis of RNA-seq replicates of WT and *IκBα*$^{-/-}$ VECs, VSMCs, and MSCs under basal and TNFα-treated conditions. Pearson correlation coefficient (R) is presented.
b) Overlapping of upregulated (left) and downregulated (right) genes in *IκBα*$^{-/-}$ VECs, VSMCs, and MSCs under basal condition.
c) GO enrichment analysis of differentially expressed genes between WT and *IκBα*$^{-/-}$ in VECs under basal condition. Enriched GO terms of upregulated and downregulated genes are marked in red and green color, respectively.
d) GO enrichment analysis of differentially expressed genes between WT and *IκBα*$^{-/-}$ in VSMCs under basal condition.
e) GO enrichment analysis of differentially expressed genes between WT and *IκBα*$^{-/-}$ in MSCs under basal condition.

Figure S1

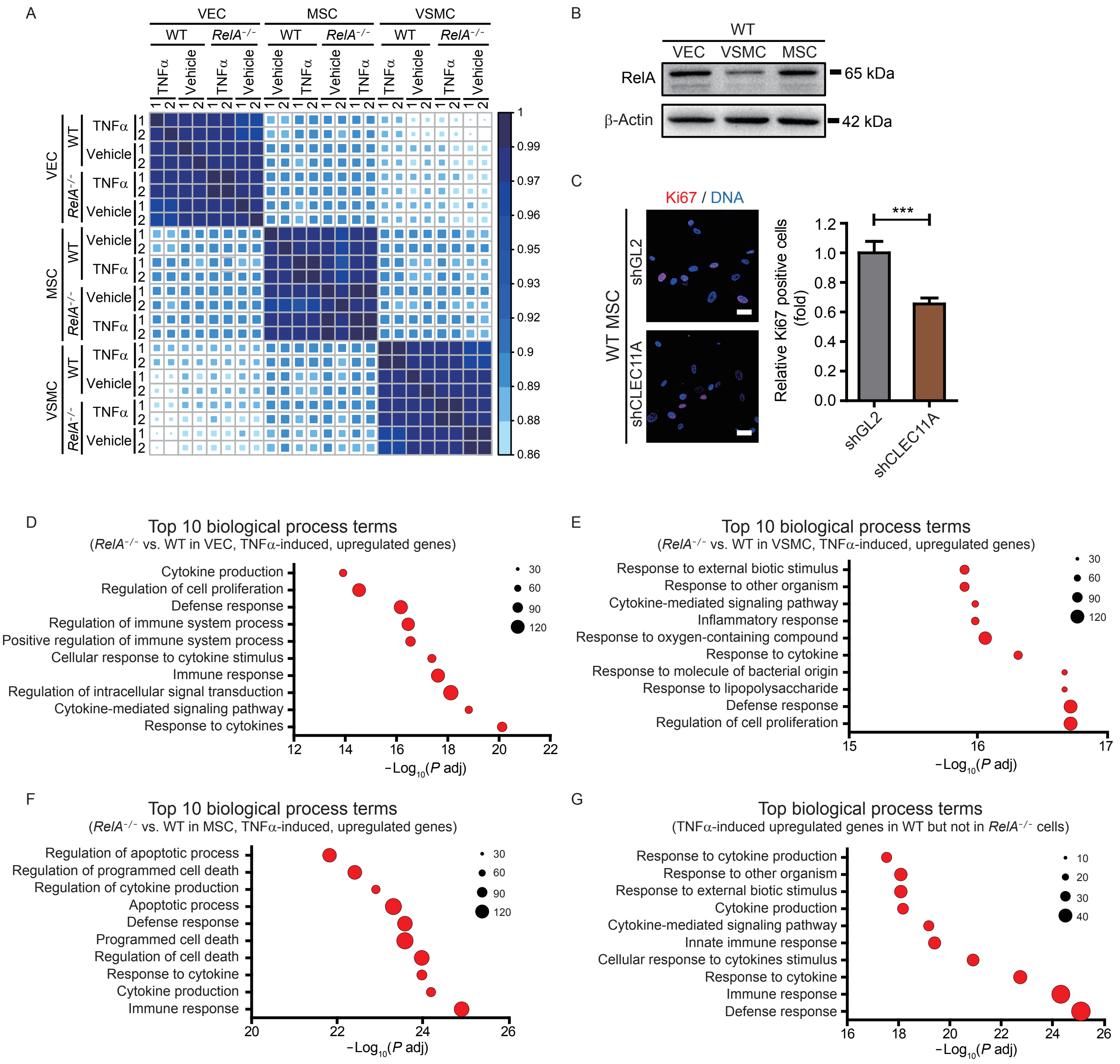
A
VEC
MSC
VSMC
WT
RelA-/-
TNFα
Vehicle
1
0.99
0.97
0.96
0.95
0.93
0.92
0.9
0.89
0.88
0.86
B
WT
VEC VSMC MSC
RelA
β-Actin
65 kDa
42 kDa
C
Ki67 / DNA
WT MSC
shGL2
shCLEC11A
***
Relative Ki67 positive cells (fold)
1.2
1.0
0.8
0.6
0.4
0.2
0.0
D
Top 10 biological process terms
(RelA-/- vs. WT in VEC, TNFα-induced, upregulated genes)
Cytokine production
Regulation of cell proliferation
Defense response
Regulation of immune system process
Positive regulation of immune system process
Cellular response to cytokine stimulus
Immune response
Regulation of intracellular signal transduction
Cytokine-mediated signaling pathway
Response to cytokines
30
60
90
120
12 14 16 18 20 22
-Log10(P adj)
E
Top 10 biological process terms
(RelA-/- vs. WT in VSMC, TNFα-induced, upregulated genes)
Response to external biotic stimulus
Response to other organism
Cytokine-mediated signaling pathway
Inflammatory response
Response to oxygen-containing compound
Response to cytokine
Response to molecule of bacterial origin
Response to lipopolysaccharide
Defense response
Regulation of cell proliferation
30
60
90
120
15 16 17
-Log10(P adj)
F
Top 10 biological process terms
(RelA-/- vs. WT in MSC, TNFα-induced, upregulated genes)
Regulation of apoptotic process
Regulation of programmed cell death
Regulation of cytokine production
Apoptotic process
Defense response
Programmed cell death
Regulation of cell death
Response to cytokine
Cytokine production
Immune response
30
60
90
120
20 22 24 26
-Log10(P adj)
G
Top biological process terms
(TNFα-induced upregulated genes in WT but not in RelA-/- cells)
Response to cytokine production
Response to other organism
Response to external biotic stimulus
Cytokine production
Cytokine-mediated signaling pathway
Innate immune response
Cellular response to cytokines stimulus
Response to cytokine
Immune response
Defense response
10
20
30
40
16 18 20 22 24 26
-Log10(P adj)

Figure S2

A

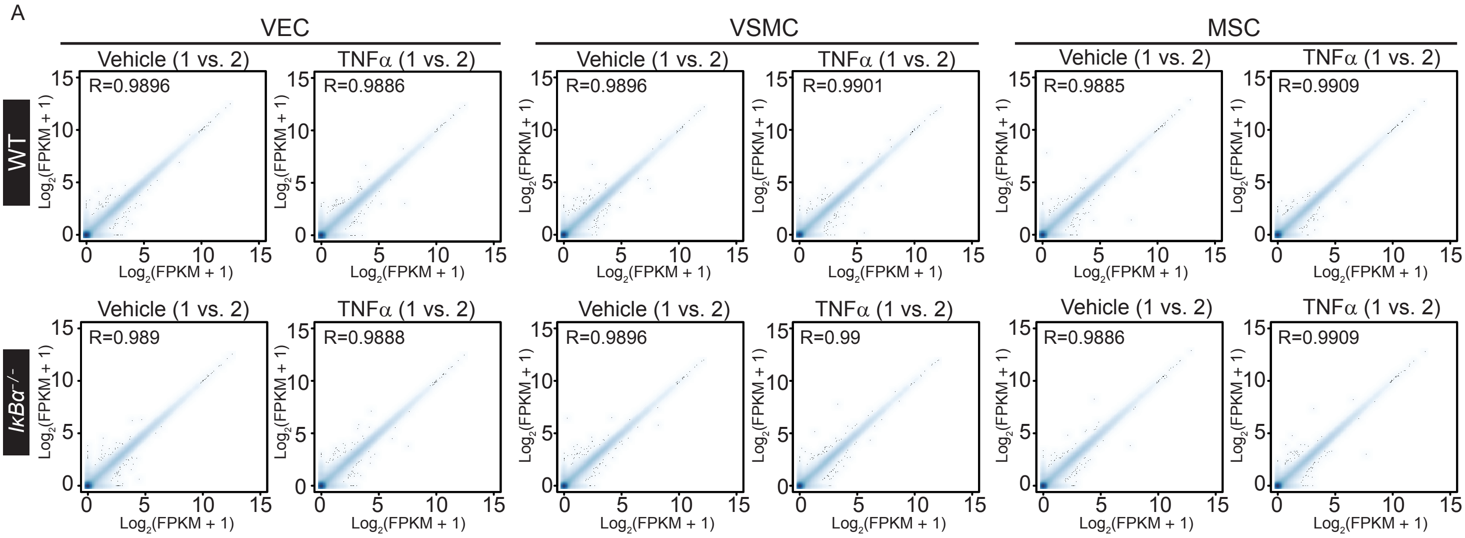

B

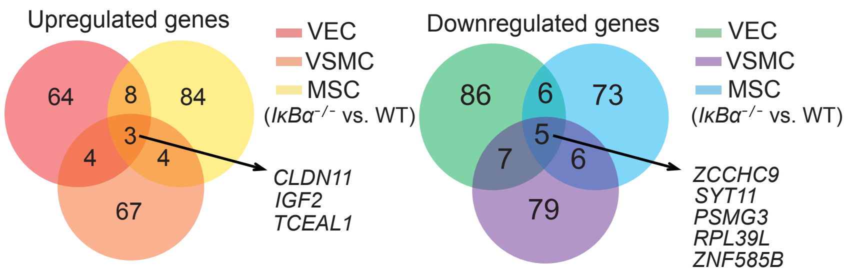

C

Top GO terms of biological process in VEC
(*IκBα*$^{-/-}$ vs. WT, vehicle)

Biological process	*P* value (adj)
Extracellular matrix organization (14)	1.59E−7
Positive regulation of leukocyte migration (7)	4.94E−4
Positive regulation of chemotaxis (7)	4.94E−4
Collagen biosynthetic process (5)	4.94E−4
Integrin-mediated signaling pathway (9)	6.01E−6
Endothelial cell differentiation (7)	1.56E−3
Smooth muscle cell migration (6)	2.35E−3
Endothelium development (7)	2.35E−3

D

Top GO terms of biological process in VSMC
(*IκBα*$^{-/-}$ vs. WT, vehicle)

Biological process	*P* value (adj)
Extracellular matrix organization (12)	9.02E−6
Response to muscle stretch (5)	6.86E−5
Tube morphogenesis (12)	1.21E−4
Response to TGFβ (10)	1.21E−4
Epithelial tube morphogenesis(11)	1.47E−4

E

Top GO terms of biological process in MSC
(*IκBα*$^{-/-}$ vs. WT, vehicle)

Biological process	*P* value (adj)
Angiogenesis (18)	7.68E−8
Positive regulation of locomotion (16)	3.71E−6
Response to lipopolysaccharide (13)	3.71E−6
Chemokine-mediated signaling pathway (8)	3.71E−6
Extracellular matrix organization (11)	6.92E−7
